# Supplementary material for: Delta Variant with P681R Critical Mutation Revealed by Ultra-Large Atomic-Scale Ab Initio Simulation: Implications for the Fundamentals of Biomolecular Interactions
Source: Viruses. 2022 Feb 24;14(3):465. doi: 10.3390/v14030465 (PMC8955942; doi:10.3390/v14030465)
Supplement: Supplementary file 1 [file viruses-14-00465-s001.zip › viruses-1591977-supplementary.pdf]

Supplementary Materials for

Delta variants with P681R critical mutation revealed by ultra-large atomic-scale *ab initio* simulation: Implications for the fundamentals of biomolecular interactions

Puja Adhikari[1], Bahaa Jawad[1,2], Praveen Rao[3], Rudolf Podgornik[4,5,6], and Wai-Yim Ching[1]

1. Department of Physics and Astronomy, University of Missouri-Kansas City, Kansas City, Missouri, 64110 USA
2. Department of Applied Sciences, University of Technology, Baghdad 10066, Iraq
3. Department of Health Management and Informatics, Department of Electrical Engineering and Computer Science, University of Missouri-Columbia, Columbia, Missouri, 65212 USA
4. School of Physical Sciences and Kavli Institute of Theoretical Science, University of Chinese Academy of Sciences, Beijing 100049, China
5. CAS Key Laboratory of Soft Matter Physics, Institute of Physics, Chinese Academy of Sciences, Beijing 100090, China
6. Wenzhou Institute of the University of Chinese Academy of Sciences, Wenzhou, Zhejiang 325000, China.
7. Department of Physics, Faculty of Mathematics and Physics, University of Ljubljana, SI-1000 Ljubljana, Slovenia

S1. COMPUTATIONAL METHODS.

S1.1 Vienna *ab initio* simulation package (VASP). The initial structure for the WT and mutation models mentioned in Section 2.1 are then optimized by using the *Vienna ab initio simulation package* (VASP), which is known for its efficiency in structural relaxation based on the *density functional theory* (DFT). We use the projector augmented wave (PAW) method with Perdew-Burke-Ernzerhof (PBE) exchange correlation functional [1] within the generalized gradient approximation (GGA). Our experience and detailed tests suggest the use of the following input parameters for biomolecular systems as more than adequate: energy cut-off 500 eV; electronic convergence of 10^{-3} eV; force convergence criteria for ionic steps at -10^{-2} eV/Å; and single k-point sampling. Details of the test is shown in **Table S2** and discussed in the **Results section 4**. All structure optimizations using VASP have been carried out at the National Energy Research Scientific Computing (NERSC) facility using Cori at the Lawrence Berkeley Laboratory or at the Research Computing Support Services (RCSS) of the University of Missouri System. The VASP-optimized structure is used as the input for the electronic structure and interatomic bonding calculations described below.

S1.2 Orthogonalized linear combination of atomic orbitals (OLCAO) method. For the electronic structure and interatomic interactions in biomolecules, we use a very different DFT method, the all-electron *orthogonalized linear combination of atomic orbitals* (OLCAO) method developed in-house. The efficacy of using the combination of these two different DFT codes is well documented [2-15]. The key feature of the OLCAO method is the provision for the effective charge (Q^*) on each atom and the *bond order* (BO) values $\rho_{\alpha\beta}$ between any pairs of atoms. They are obtained from the *ab initio* wave functions with atomic basis expansion calculated quantum mechanically

$$Q_\alpha^* = \sum_i \sum_{m,occ} \sum_{j,\beta} C_{i\alpha}^{*m} C_{j\beta}^m S_{i\alpha,j\beta} \quad (1)$$

$$\rho_{\alpha\beta} = \sum_{m,occ} \sum_{i,j} C_{i\alpha}^{*m} C_{j\beta}^{m} S_{i\alpha,j\beta} \quad (2)$$

In the above equations, $S_{i\alpha,j\beta}$ are the overlap integrals between the ith orbital in α^{th} atom and the jth orbital in the βth atom. $C_{j\beta}^{m}$ are the eigenvector coefficients of the m^{th}occupied molecular orbital. The *partial charge* (PC) or ($\Delta Q_\alpha = Q_\alpha^0 - Q_\alpha^*$) is the deviation of the effective charge Q_α^* from the neutral atomic charge Q_α^0 on the same atom α. The BO quantifies the strength of the bond between two atoms and usually scales with the *bond length* (BL) but is also influenced by the surrounding atoms. The calculations of PC and BO is based on the Mulliken scheme [16, 17], hence are basis-dependent, so that comparisons of BO values using different basis sets or different DFT methods should be treated with caution. We use a one-point calculation to characterize the bonding rather than traditional energy (or enthalpy) difference calculation (two-point calculation) which requires reference energy for comparison and depends on the method used. The sum of all BO values within a single structure unit gives the *total bond order* (TBO). This is an innovative aspect of our method in the study of complex biomolecular materials capable to yield a large amount of crucially important data.

S2. ADDITIONAL SUPPORTING INFORMATION.

(a) We have added the AABP data for a single mutation D950N in domain HR1-CH and listed in **Table S8**. The AABP data essentially confirm the observations listed in the previous **section 4.3** that mutation reduces the AABP values and weakens the bonding between amino acids. Both WT and mutated cases have the same number of NL interactions (see **Figure S7**). However, the bonding in each case is different as shown in **Table S9.** Comparing **Figure S7** for mutation D950N in HR1-CH with the main **Figure 4** for mutations D614G and P681R in SD2-FP, we can clearly see that (1) The overall shape and size of WT D950 have very small difference with the mutated N950; (2) These results are very different from mutations D614G and P681R in SD2-FP in **Figure 4**; (3) The PC of WT D950 and its interacting group (+0.209e$^-$) is significantly increased in case of mutated N950 and its interacting group(+1.146e$^-$). These observations again support our assertion that the effect on mutation critically depends on the location of the targeted residue and its interactions with its neighboring AAs, NN and NL ones, via the detailed atomic scale calculation of AABP values.

(b) We have PC for each AAs of WT HR1-CH listed in **Table S4** and bar graph in **Figure S6**. **Figure S6** shows PC of AA D950 changes from highly negative -0.813 e$^-$ to 0.073 e$^-$ when mutates to N950.

Supporting Figures:

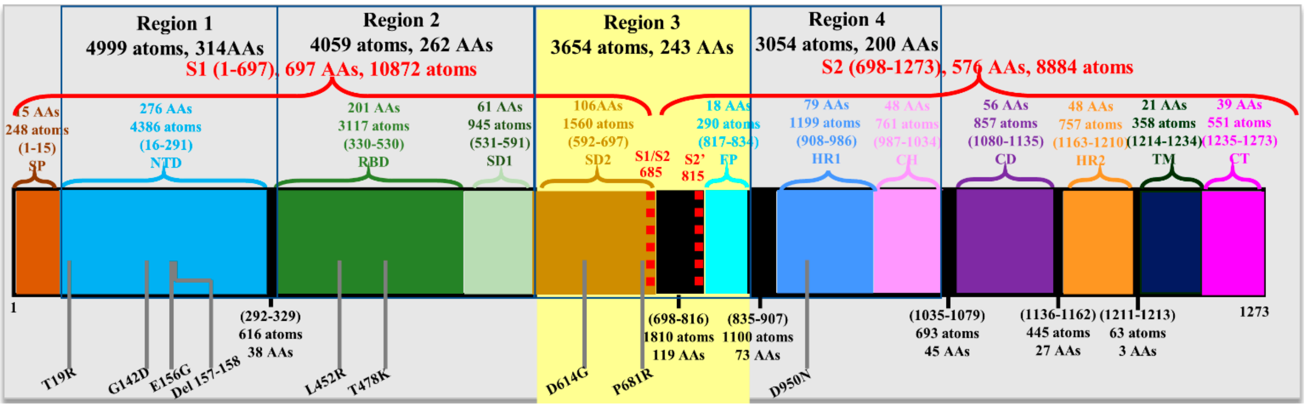

Figure S1. Detailed schematic of S-protein in SARS-CoV-2 colored by domains: SP, NTD, RBD, SD2, furin cleavage site (S1/S2), FP, HR1, CH, CD, HR2, TM, and CT. The delta variant mutation sites are marked by gray solid line in the bottom. In the present work, our calculations have been mainly carried out on region 3 of SD2 and FP domains.

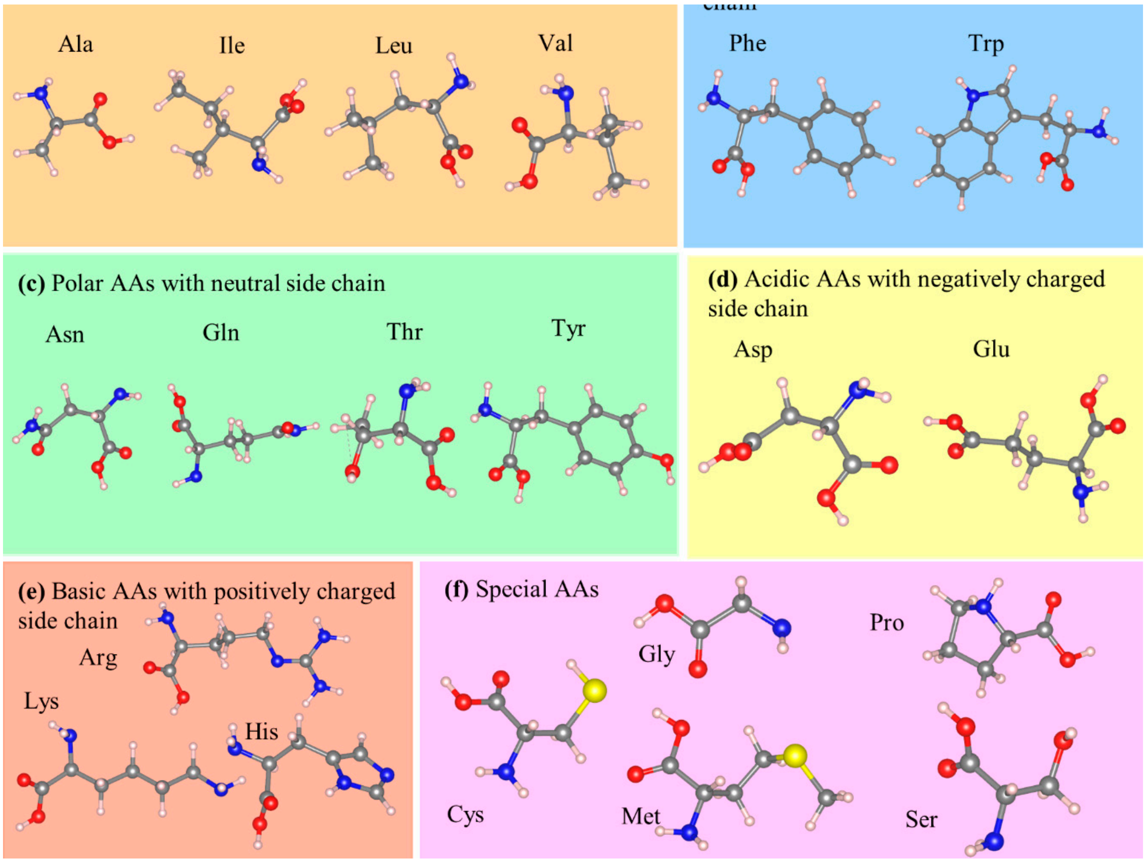

Figure S2. The 20 amino acids in **Table S1** are divided into 6 functional groups based on the nature of their sidechains: (a) and (b) for hydrophobic AAs, (c) for neutral polar AAs, (d) and (e) for charged polar AAs, and (f) for unique AAs.

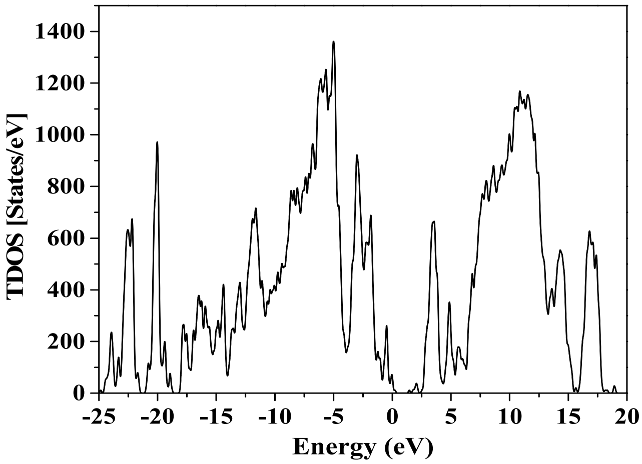

Figure S3. Calculated total density of stated (TDOS) for WT SD2-FP model.

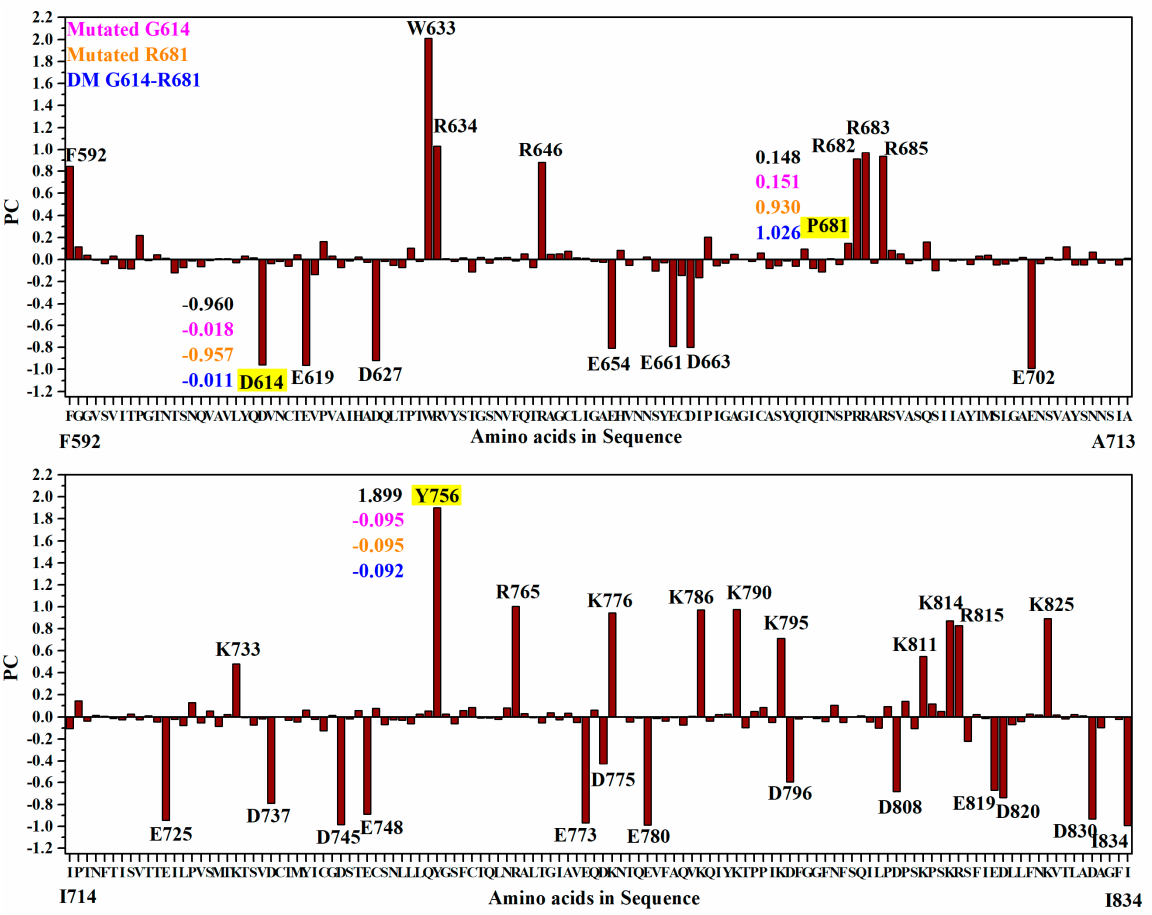

Figure S4. Bar graph with PC distribution for WT SD2-FP. PC values are marked for the two mutation sites 614 and 681 showing values in different color for different mutation cases.

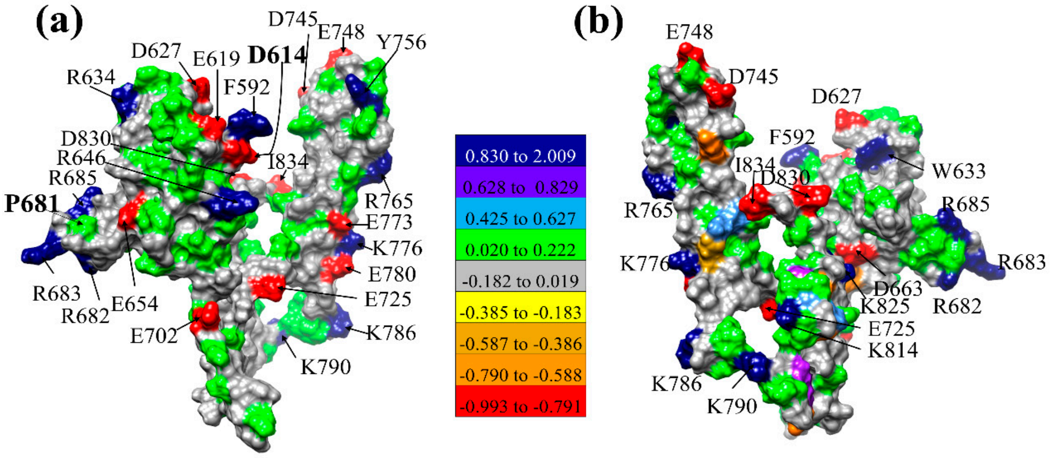

Figure S5. (a) Comparison of PC on the solvent excluded surface between P681 and D614. **(b)** 180° orientation of **(a)**.

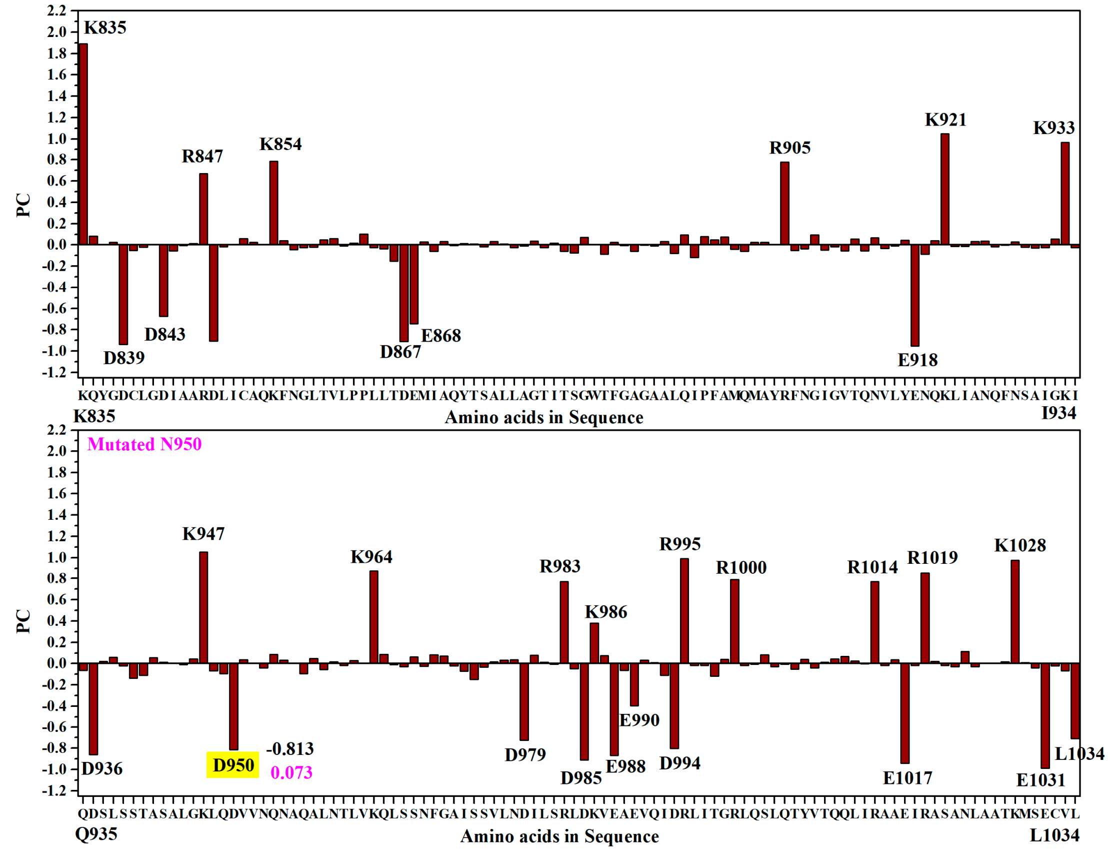

Figure S6. Bar graph with PC distribution for WT HR1-CH. PC values are marked for the two mutation sites 950 showing values in different color for D950N mutation.

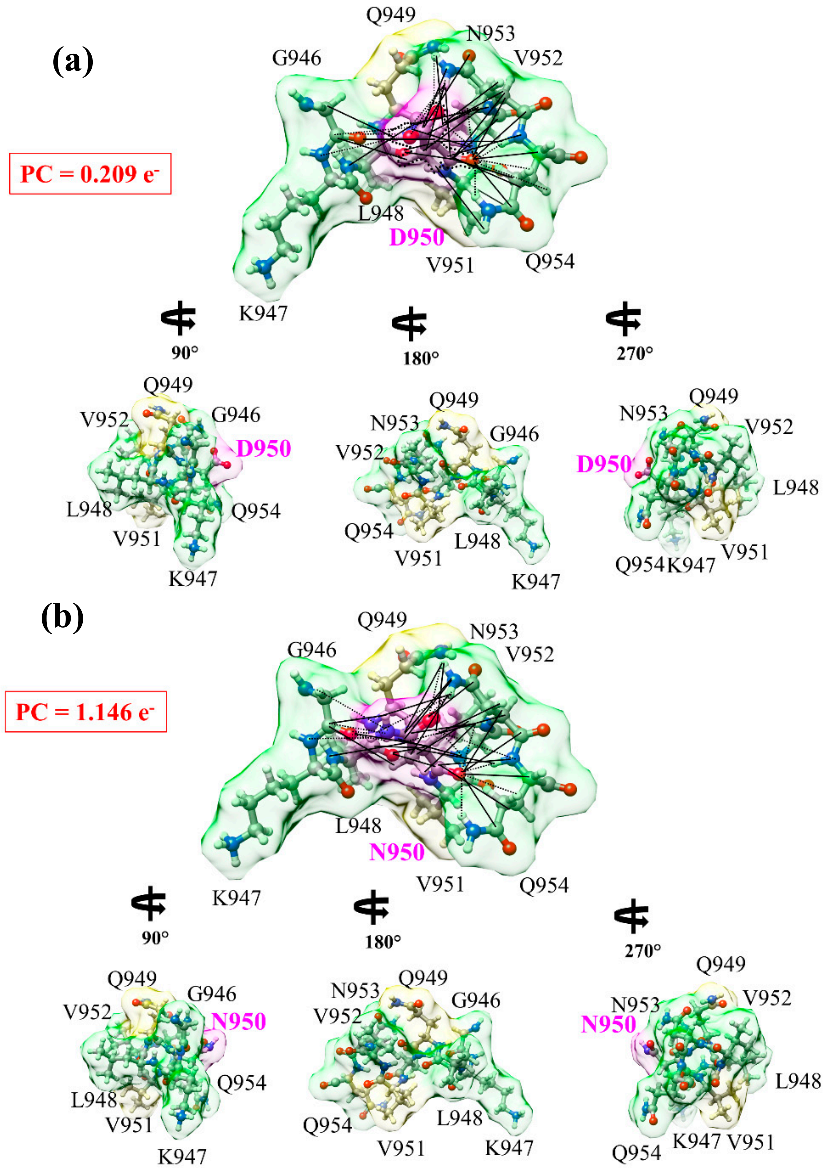

Figure S7. Interactions of 950th site in two HR1-CH models in Delta variant: (a) WT D950 and (b) mutated N950. In each case, the ball and stick sketch of all participating AAs are shown (red, O, grey, C, blue N, white, H). The focused 950th AA is marked light pink. Its two NNs are marked light yellow, and its interacting NL AAs are marked light green. All NL interactions are marked by solid lines and dashed lines show HBs. All these NL interactions with the bonds formed are shown in **Table S9.** At the lower part of each of the figure, three smaller figures show the same figure rotated for 90˚, 180˚, 270˚ form left to right. These figures show some of the most detailed information on the AA-AA interaction at atomic scale. In the same figure, the partial charge on each group is shown in the red boxes. These PC values are obtained summing PC values of individual AAs in each interacting group. The mutated case has distinctly higher positive PC.

Supporting Tables:

Table S1. 20 canonical amino acids in alphabetical order. The last column shows their functional group.

No.	Amino acid	3 letter code	1 letter code	TBO	No of Atoms	TBO /Atom	No. of bonds	No. of HB	Group
1	**Alanine**	**Ala**	**A**	4.380	13	0.337	25	5	a
2	**Arginine**	**Arg**	**R**	9.434	26	0.363	52	12	e
3	**Asparagine**	**Asn**	**N**	6.016	17	0.354	30	7	c
4	**Asparatic**	**Asp**	**D**	5.456	16	0.341	33	7	d
5	**Cystein**	**Cys**	**C**	4.512	14	0.322	23	3	f
6	**Glutamine**	**Gln**	**Q**	7.224	20	0.361	38	9	c
7	**Glutamic acid**	**Glu**	**E**	6.664	19	0.351	37	7	d
8	**Glycine**	**Gly**	**G**	3.198	10	0.320	16	4	f
9	**Histidine**	**His**	**H**	8.164	20	0.408	41	7	e
10	**Isoleucin**	**Ile**	**I**	8.033	22	0.365	43	6	a
11	**Leucin**	**Leu**	**L**	8.032	22	0.365	40	4	a
12	**Lysine**	**Lys**	**K**	8.771	24	0.365	44	7	e
13	**Methonine**	**Met**	**M**	6.879	20	0.344	35	2	f
14	**Phenylalanine**	**Phe**	**F**	10.119	23	0.440	47	3	b
15	**Proline**	**Pro**	**P**	6.488	17	0.382	42	6	f
16	**Serine**	**Ser**	**S**	4.481	14	0.320	27	4	f
17	**Threonine**	**Thr**	**T**	5.642	17	0.332	31	5	c
18	**Tryptophan**	**Trp**	**W**	12.436	27	0.461	53	2	b
19	**Tyrosine**	**Tyr**	**Y**	10.248	24	0.427	47	3	c
20	**Valine**	**Val**	**V**	6.758	19	0.356	36	4	a

Table S2. Four SD2-FP models ((a) to (d)) and two HR1-CH models ((e) and (f)) with the number of atoms, total energy and time used in Cori.

Models	Atoms	Total Energy (eV)	Convergence	Time in Cori (node hour)
(a) WT P681 & D614	3654	-22293.12593	-0.0479275	12x48x64= 36864
(b) Mutated R681	3664	-22350.51693	-0.0339289	13x48x64= 39936
(c) Mutated G614	3649	-22259.50298	-0.0339803	13x48x64= 39936
(d) DM G614-R681	3659	-22316.36542	-0.0324175	12x48x64=36864
(e) WT D950	3054	-18528.45614	-0.00613611	6x48x64= 18432
(f) Mutated N950	3056	-18538.10922	-0.00622162	5x48x64=15360

Table S3: List of PC value for each amino acids with their sequence number for WT SD2-FP.

AA Seq No	PC	AA Seq No	PC	AA Seq No	PC	AA Seq No	PC	AA Seq No	PC
F592	0.843	N641	0.017	Q690	0.158	T739	-0.030	I788	0.021
G593	0.116	V642	0.021	S691	-0.099	M740	-0.046	Y789	0.027
G594	0.040	F643	-0.013	I692	0.005	Y741	0.061	K790	0.977
V595	-0.003	Q644	0.051	I693	-0.013	I742	-0.022	T791	-0.098
S596	-0.033	T645	-0.070	A694	-0.003	C743	-0.123	P792	0.050
V597	0.032	R646	0.878	Y695	-0.042	G744	0.013	P793	0.085
I598	-0.080	A647	0.047	T696	0.034	D745	-0.986	I794	-0.050
T599	-0.084	G648	0.051	M697	0.042	S746	-0.018	K795	0.711
P600	0.218	C649	0.074	S698	-0.048	T747	0.060	D796	-0.595
G601	-0.008	L650	0.016	L699	-0.040	E748	-0.889	F797	-0.018
T602	0.046	I651	0.013	G700	-0.013	C749	0.079	G798	-0.003
N603	0.011	G652	-0.017	A701	0.021	S750	-0.069	G799	-0.015
T604	-0.118	A653	-0.024	E702	-0.991	N751	-0.025	F800	-0.041
S605	-0.070	E654	-0.808	N703	-0.035	L752	-0.030	N801	0.104
N606	-0.013	H655	0.085	S704	0.020	L753	-0.061	F802	-0.050
Q607	-0.065	V656	-0.051	V705	-0.004	L754	0.027	S803	-0.001
V608	-0.009	N657	0.000	A706	0.114	Q755	0.053	Q804	0.012
A609	0.010	N658	0.022	Y707	-0.046	Y756	1.899	I805	-0.046
V610	0.009	S659	-0.103	S708	-0.046	G757	0.026	L806	-0.099
L611	-0.029	Y660	-0.029	N709	0.066	S758	-0.061	P807	0.092
Y612	0.032	E661	-0.790	N710	-0.033	F759	0.057	D808	-0.680
Q613	0.016	C662	-0.144	S711	-0.005	C760	0.085	P809	0.143
D614	-0.960	D663	-0.799	I712	-0.047	T761	-0.009	S810	-0.104
V615	-0.036	I664	-0.162	A713	0.013	Q762	-0.008	K811	0.549
N616	-0.017	P665	0.204	I714	-0.104	L763	-0.021	P812	0.119
C617	-0.058	I666	-0.055	P715	0.145	N764	0.084	S813	0.049
T618	0.045	G667	-0.031	T716	-0.039	R765	1.006	K814	0.871
E619	-0.962	A668	0.047	N717	0.016	A766	0.029	R815	0.827
V620	-0.134	G669	0.004	F718	0.006	L767	-0.006	S816	-0.221
P621	0.165	I670	-0.015	T719	-0.014	T768	-0.053	F817	0.021
V622	0.032	C671	0.062	I720	-0.024	G769	0.040	I818	-0.014
A623	-0.071	A672	-0.077	S721	0.027	I770	-0.024	E819	-0.669
I624	-0.012	S673	-0.054	V722	-0.024	A771	0.035	D820	-0.739
H625	0.023	Y674	-0.012	T723	0.011	V772	-0.050	L821	-0.068
A626	-0.024	Q675	-0.061	T724	-0.044	E773	-0.967	L822	-0.043
D627	-0.918	T676	0.098	E725	-0.945	Q774	0.062	F823	0.026
Q628	-0.017	Q677	-0.080	I726	-0.020	D775	-0.427	N824	0.018
L629	-0.051	T678	-0.110	L727	-0.079	K776	0.941	K825	0.890
T630	-0.071	N679	0.007	P728	0.128	N777	-0.003	V826	0.018
P631	0.103	S680	-0.044	V729	-0.055	T778	-0.045	T827	-0.017
T632	-0.014	P681	0.148	S730	0.056	Q779	-0.009	L828	0.022
W633	2.009	R682	0.911	M731	-0.083	E780	-0.987	A829	0.012
R634	1.033	R683	0.971	T732	0.022	V781	-0.014	D830	-0.934
V635	0.009	A684	-0.030	K733	0.481	F782	-0.039	A831	-0.099
Y636	-0.015	R685	0.936	T734	-0.005	A783	-0.006	G832	0.003
S637	0.015	S686	0.086	S735	-0.072	Q784	-0.075	F833	-0.023
T638	-0.112	V687	0.054	V736	-0.016	V785	0.005	I834	-0.993
G639	0.022	A688	-0.037	D737	-0.790	K786	0.975		
S640	-0.031	S689	-0.006	C738	0.004	Q787	-0.038		

Table S4: List of PC value for each amino acids with their sequence number for WT HR1-CH.

AA Seq No	PC	AA SeqNo	PC	AA SeqNo	PC	AA SeqNo	PC
K835	1.893	G885	0.072	Q935	-0.065	D985	-0.909
Q836	0.082	W886	0.003	D936	-0.861	K986	0.381
Y837	0.002	T887	-0.087	S937	0.022	V987	0.074
G838	0.025	F888	0.025	L938	0.058	E988	-0.867
D839	-0.939	G889	-0.007	S939	-0.022	A989	-0.066
C840	-0.054	A890	-0.061	S940	-0.138	E990	-0.396
L841	-0.022	G891	-0.001	T941	-0.110	V991	0.032
G842	0.003	A892	-0.011	A942	0.054	Q992	0.011
D843	-0.675	A893	0.031	S943	0.014	I993	-0.111
I844	-0.056	L894	-0.080	A944	0.004	D994	-0.803
A845	-0.007	Q895	0.095	L945	-0.012	R995	0.991
A846	0.013	I896	-0.117	G946	0.044	L996	-0.017
R847	0.672	P897	0.079	K947	1.051	I997	-0.016
D848	-0.905	F898	0.047	L948	-0.067	T998	-0.117
L849	-0.017	A899	0.074	Q949	-0.093	G999	0.039
I850	0.006	M900	-0.042	D950	-0.813	R1000	0.789
C851	0.061	Q901	-0.061	V951	0.038	L1001	-0.016
A852	0.024	M902	0.024	V952	0.002	Q1002	-0.007
Q853	0.000	A903	0.023	N953	-0.039	S1003	0.084
K854	0.787	Y904	0.000	Q954	0.088	L1004	-0.031
F855	0.040	R905	0.777	N955	0.032	Q1005	-0.006
N856	-0.044	F906	-0.054	A956	0.001	T1006	-0.054
G857	-0.024	N907	-0.036	Q957	-0.096	Y1007	0.039
L858	-0.023	G908	0.093	A958	0.046	V1008	-0.040
T859	0.047	I909	-0.049	L959	-0.055	T1009	0.011
V860	0.060	G910	-0.019	N960	0.019	Q1010	0.044
L861	-0.010	V911	-0.055	T961	-0.018	Q1011	0.066
P862	0.016	T912	0.056	L962	0.030	L1012	0.026
P863	0.104	Q913	-0.055	V963	0.001	I1013	-0.004
L864	-0.024	N914	0.068	K964	0.874	R1014	0.770
L865	-0.037	V915	-0.034	Q965	0.085	A1015	-0.017
T866	-0.153	L916	-0.012	L966	-0.010	A1016	0.037
D867	-0.912	Y917	0.042	S967	-0.028	E1017	-0.943
E868	-0.745	E918	-0.954	S968	0.064	I1018	-0.018
M869	0.030	N919	-0.086	N969	-0.025	R1019	0.857
I870	-0.060	Q920	0.039	F970	0.082	A1020	0.021
A871	0.034	K921	1.050	G971	0.071	S1021	-0.017
Q872	-0.005	L922	-0.014	A972	-0.020	A1022	-0.029
Y873	0.011	I923	-0.013	I973	-0.071	N1023	0.115
T874	0.008	A924	0.032	S974	-0.151	L1024	-0.029
S875	-0.017	N925	0.035	S975	-0.033	A1025	0.001
A876	0.031	Q926	-0.016	V976	0.016	A1026	0.000
L877	0.008	F927	-0.003	L977	0.033	T1027	0.016
L878	-0.027	N928	0.029	N978	0.035	K1028	0.977
A879	-0.012	S929	-0.022	D979	-0.727	M1029	0.008
G880	0.035	A930	-0.029	I980	0.079	S1030	-0.043
T881	-0.027	I931	-0.028	L981	0.014	E1031	-0.990
I882	0.018	G932	0.056	S982	-0.006	C1032	-0.021
T883	-0.059	K933	0.968	R983	0.772	V1033	-0.070
S884	-0.075	I934	-0.025	L984	-0.047	L1034	-0.709

Table S5: NL bonding information for WT SD2-FP shown in **Figure 4(a)** and **4(b)**.

**P681**	**AA1**	**AA2**	**BL**	**BO**
C-C	P681(C)	R683(CA)	4.3112	0.0003
C-O	P681(C)	N679(O)	3.8317	0.0016
C-O	A684(CA)	P681(O)	3.5811	0.0015
C-O	R683(C)	P681(O)	3.9893	0.0012
C-O	R685(CA)	P681(O)	4.0486	0.0012
C-O	A684(C)	P681(O)	3.7966	0.0011
C-O	P681(CA)	N679(O)	3.9701	0.0009
C-O	R683(CA)	P681(O)	4.1355	0.0006
C-O	P681(C)	R685(O)	4.0265	0.0001
H-C	R685(H)	P681(CA)	4.3914	0.0005
H-C	N679(HB2)	P681(CG)	4.4225	0.0002
H-C	A684(H)	P681(CA)	3.7109	0.0001
H-C	P681(HB2)	A688(C)	4.1737	0.0001
H-C	A688(HA)	P681(CA)	4.3491	0.0001
H-H	N679(HB2)	P681(HD3)	3.6204	0.0001
H-H	N679(HB2)	P681(HG2)	4.1921	0.0001
H-H	P681(HB2)	A688(HB1)	4.4138	0.0001
N...H	R683(N)	P681(HA)	4.3121	0.0002
N...H	P681(N)	N679(HB2)	4.4207	0.0001
N-C	R685(N)	P681(C)	4.2318	0.0018
O...H	A684(H)	P681(O)	1.9853	0.0221
O...H	R685(H)	P681(O)	2.0272	0.0195
O...H	A684(HB3)	P681(O)	4.1600	0.0001
**D614**				
C-O	G593(C)	D614(OD1)	3.8390	0.0008
C-O	D614(CG)	F592(O)	4.1658	0.0001
C-O	D614(C	F592(O)	4.4372	0.0001
C-O	F592(C)	D614(OD1)	4.4956	0.0001
H-C	G593(HA3)	D614(CA)	4.1604	0.0002
H-C	N616(H)	D614(C)	4.3890	0.0001
H-C	D614(H)	G593(C)	4.4405	0.0001
N...H	D614(N)	G648(HA3)	4.2644	0.0002
N-C	N616(N)	D614(C)	4.2832	0.0002
N-O	G593(N)	D614(OD1)	4.1450	0.0011
O...H	G593(HA2)	D614(OD1)	2.3697	0.0006
O...H	G594(H)	D614(OD1)	2.8758	0.0009
O...H	N616(H)	D614(O)	4.0740	0.0001
O...H	D614(HB3)	F592(O)	4.1371	0.0001

Table S6: NL bonding information for mutated R681 shown in **Figure 4(c)** and **4(d)**.

**R681**	**AA1**	**AA2**	**BL**	**BO**
C-C	E654(CD)	R681(CZ)	4.4034	0.0007
C-C	N679(C)	R681(CA)	4.3343	0.0002
C-C	R681(C)	R683(CA)	4.4096	0.0003
C-O	R681(CZ)	E654(OE1)	3.9477	0.0016
C-O	R681(C)	N679(O)	3.8731	0.0015
C-O	R681(CA)	N679(O)	3.9977	0.0007
C-O	R683(C)	R681(O)	4.1159	0.0009
C-O	R683(CA)	R681(O)	4.2376	0.0005
C-O	A684(CA)	R681(O)	3.6842	0.0012
C-O	A684(C)	R681(O)	3.8998	0.0008
C-O	R685(CA)	R681(O)	4.1073	0.0010
H-C	R681(HH12)	E654(CG)	3.9178	0.0050
H-C	R681(HH11)	E654(CD)	3.8786	0.0006
H-C	R681(HB3)	A688(CB)	4.0259	0.0002
H-C	R681(HB2)	A688(C)	4.0362	0.0001
H-C	A684(H)	R681(CA)	3.8994	0.0003
H-C	A688(HB2)	R681(CG)	4.4072	0.0002
H-C	A688(HA)	R681(CA)	4.0814	0.0001
H-H	R681(HB3)	A688(HB2)	3.6654	0.0002
H-H	R681(HB2)	A688(HB1)	4.2281	0.0002
H-H	R681(HB3)	A688(HB3)	4.1173	0.0001
N...H	R683(N)	R681(HA)	4.4977	0.0001
N-C	R685(N)	R681(C)	4.3002	0.0013
N-O	R681(NH2)	E654(OE1)	4.1715	0.0002
N-O	R681(NH2)	E654(OE2)	4.2740	0.0001
O...H	R681(HH12)	E654(OE1)	1.8646	0.0579
O...H	R681(HH12)	E654(OE2)	2.3609	0.0012
O...H	R681(HH11)	A688(O)	3.3227	0.0001
O...H	R681(HH11)	E654(OE2)	3.6317	0.0003
O...H	R681(HB2)	A688(O)	3.7363	0.0001
O...H	A684(H)	R681(O)	2.1127	0.0161
O...H	A684(HB3)	R681(O)	4.2097	0.0001
O...H	R685(H)	R681(O)	2.1173	0.0167
**G614**				
C-O	F592(C)	G614(O)	4.3773	0.0001
C-O	G614(CA)	Y612(O)	4.4818	0.0002
H-C	G614(H)	Y612(CA)	4.1262	0.0002

Table S7: NL bonding information for double mutation R681 & G614 shown in **Figure 4(e)** and **4(f)**.

**R681**	**AA1**	**AA2**	**BL**	**BO**
C-C	N679(C)	R681(CA)	4.2988	0.0002
C-C	R681(C)	R683(CA)	4.4065	0.0002
C-O	R681(C)	N679(O)	3.7966	0.0018
C-O	R681(CA)	N679(O)	3.9376	0.0008
C-O	R681(C)	R685(O)	3.9700	0.0001
C-O	R683(C)	R681(O)	4.1225	0.0009
C-O	R683(CA)	R681(O)	4.2607	0.0004
C-O	A684(CA)	R681(O)	3.6620	0.0011
C-O	A684(C)	R681(O)	3.8577	0.0009
C-O	R685(CA)	R681(O)	4.0559	0.0011
H-C	R681(HB3)	A688(CB)	4.0901	0.0002
H-C	R681(HB2)	A688(C)	4.1631	0.0001
H-C	R681(HH11)	E654(CD)	4.2840	0.0001
H-C	A684(H)	R681(CA)	3.8290	0.0002
H-C	R685(H)	R681(CA)	4.4735	0.0005
H-C	A688(HA)	R681(CA)	4.1218	0.0001
H-H	R681(HB3)	A688(HB2)	3.7532	0.0001
H-H	R681(HB3)	A688(HB3)	4.1294	0.0001
H-H	R681(HB2)	A688(HB1)	4.3108	0.0001
O…H	R681(HH12)	E654(OE1)	2.7545	0.0025
O…H	R681(HH12)	E654(OE2)	3.0339	0.0002
O…H	A684(H)	R681(O)	2.1218	0.0149
O…H	A684(HB1)	R681(O)	2.8768	0.0001
O…H	A684(HB3)	R681(O)	4.1990	0.0001
O…H	R685(H)	R681(O)	2.0797	0.0188
N-C	R685(N)	R681(C)	4.2576	0.0015
N...H	R683(N)	R681(HA)	4.4463	0.0001
**G614**				
C-O	G614(CA)	Y612(O)	4.4363	0.0003
H-C	G614(H)	Y612(CA)	4.1801	0.0002
N-C	N616(N)	G614(C)	4.4429	0.0001

Table S8. AABP of two HR1-CH models.

**Models**	**Total AABP**	**NN AABP**	**Non-local AABP**	**AABP from HB**	**No. of NL AAs**	**Data notation**
WT D950	1.163	1.021	0.143	0.154	6	D950-1.163-1.021-0.143-0.154-0
Mutated N950	1.093	1.009	0.084	0.106	6	N950-1.093-1.009-0.084-0.106-1

Table S9: Bonding information for WT and mutated HR1-CH shown in **Figure S7.**

D950	BL	BO	AA1	AA2	N950	BL	BO	AA1	AA2
C-C	4.4115	0.0007	D950(C)	Q954(CG)	C-C	4.3161	0.0002	L948(C)	N950(CA)
C-C	4.4565	0.0001	D950(C)	N953(CA)	C-O	3.9113	0.0011	N950(C)	L948(O)
C-C	4.4797	0.0001	D950(C)	V952(C)	C-O	3.9991	0.0006	N950(CA)	L948(O)
C-O	3.9614	0.0009	D950(CA)	G946(O)	C-O	4.0112	0.0005	N950(CA)	G946(O)
C-O	4.0222	0.0007	D950(C)	L948(O)	C-O	3.9725	0.0007	N953(CG)	N950(OD1)
C-O	4.1299	0.0005	D950(CA)	L948(O)	C-O	3.9971	0.0008	N953(C)	N950(O)
C-O	3.6276	0.0014	N953(CG)	D950(OD1)	C-O	4.0473	0.0002	N953(CA)	N950(O)
C-O	3.8914	0.0012	N953(C)	D950(O)	C-O	3.8267	0.0010	Q954(CA)	N950(O)
C-O	3.9653	0.0003	N953(CA)	D950(O)	C-O	4.1235	0.0002	Q954(CD)	N950(O)
C-O	3.6803	0.0015	Q954(CA)	D950(O)	H-C	3.8882	0.0003	N950(HA)	N953(CA)
C-O	4.0099	0.0002	Q954(CD)	D950(O)	H-C	4.1012	0.0002	N950(HB2)	G946(C)
H-C	3.8349	0.0003	D950(HA)	N953(CA)	H-C	4.3837	0.0001	N950(HD21)	G946(C)
H-C	4.0555	0.0001	D950(HB2)	G946(C)	H-C	3.8755	0.0001	N953(HD22)	N950(CB)
H-C	4.1004	0.0001	D950(H)	G946(CA)	H-C	3.9892	0.0002	N953(HB2)	N950(CB)
H-C	4.4805	0.0001	D950(HB3)	Q954(CD)	H-C	4.0192	0.0002	N953(HB2)	N950(CG)
H-C	3.7144	0.0017	N953(HD22)	D950(CB)	H-C	4.3440	0.0001	N953(HD21)	N950(CG)
H-C	4.4845	0.0003	N953(HD22)	D950(C)	H-C	3.9659	0.0004	Q954(H)	N950(CA)
H-C	3.7387	0.0001	N953(HB2)	D950(CG)	H-C	4.0778	0.0001	Q954(HB2)	N950(C)
H-C	3.8752	0.0001	N953(HB2)	D950(CB)	H-H	3.7496	0.0002	N950(HA)	N953(HD21)
H-C	3.8121	0.0002	Q954(H)	D950(CA)	H-H	3.8780	0.0003	N950(HA)	N953(HB3)
H-C	3.9420	0.0001	Q954(HB2)	D950(C)	H-H	3.9617	0.0001	N950(HB3)	N953(HB2)
H-H	3.7331	0.0003	D950(HA)	N953(HD21)	H-H	4.0594	0.0001	N950(HD21)	N953(HD22)
H-H	3.8226	0.0003	D950(HA)	N953(HB3)	N...H	4.0470	0.0001	K947(N)	N950(HD22)
H-H	4.3138	0.0001	D950(HB3)	Q954(HE21)	N...H	4.0439	0.0002	N950(ND2)	N953(HD22)
N...H	4.1160	0.0002	K947(N)	D950(H)	N...H	4.1611	0.0003	N950(N)	N953(HB2)
N...H	3.8694	0.0001	D950(N)	N953(HD22)	N...H	4.4219	0.0001	N950(ND2)	G946(HA3)
N...H	4.1080	0.0004	D950(N)	N953(HB2)	N...H	4.4693	0.0001	V952(N)	N950(HA)
N...H	4.4338	0.0002	V952(N)	D950(HA)	N...H	4.3346	0.0001	Q954(N)	N950(HA)
N-C	4.2093	0.0011	D950(N)	G946(C)	N-C	4.0100	0.0005	N950(ND2)	G946(C)
N-C	3.8942	0.0039	N953(ND2)	D950(CG)	N-C	3.9414	0.0001	N953(ND2)	N950(CG)
N-C	3.9615	0.0012	Q954(N)	D950(C)	N-C	4.0431	0.0004	Q954(N)	N950(C)
N-N	4.4244	0.0002	L948(N)	D950(N)	N-N	4.3688	0.0002	L948(N)	N950(N)
N-N	4.4000	0.0005	D950(N)	V952(N)	N-N	4.3199	0.0001	N950(N)	N953(ND2)
N-N	4.2600	0.0001	D950(N)	N953(ND2)	N-N	4.4405	0.0004	N950(N)	V952(N)
O...H	2.0770	0.0201	D950(H)	G946(O)	O...H	2.1235	0.0172	N950(HD22)	G946(O)
O...H	1.6805	0.0686	N953(HD22)	D950(OD1)	O...H	2.3166	0.0090	N950(H)	G946(O)
O...H	3.0025	0.0004	N953(HB2)	D950(O)	O...H	2.0216	0.0254	N953(HD22)	N950(OD1)
O...H	3.3303	0.0005	N953(HD21)	D950(OD1)	O...H	2.9768	0.0006	N953(HB2)	N950(O)
O...H	3.8586	0.0011	N953(HD22)	D950(OD2)	O...H	3.5009	0.0002	N953(HD21)	N950(OD1)
O...H	1.8985	0.0321	Q954(H)	D950(O)	O...H	2.0562	0.0213	Q954(H)	N950(O)
O...H	3.6536	0.0001	Q954(HE22)	D950(O)	O...H	3.6756	0.0001	Q954(HE22)	N950(O)
O...H	4.0865	0.0003	Q954(HG2)	D950(O)	O...H	4.1025	0.0002	Q954(HG2)	N950(O)
O...H	4.3762	0.0001	Q954(HB3)	D950(O)					

REFERENCES

1. Perdew, J.P., K. Burke, and M. Ernzerhof, *Generalized gradient approximation made simple.* Physical review letters, 1996. **77**(18): p. 3865, DOI: 10.1103/PhysRevLett.77.3865.
2. Jawad, B., P. Adhikari, R. Podgornik, and W.-Y. Ching, *Key interacting residues between RBD of SARS-CoV-2 and ACE2 receptor: Combination of molecular dynamic simulation and density functional calculation.* Journal of Chemical Information and Modeling, 2021, DOI: 10.1021/acs.jcim.1c00560.
3. Ching, W.-Y., P. Adhikari, B. Jawad, and R. Podgornik, *Ultra-Large-Scale Ab Initio Quantum Chemical Computation of Bio-Molecular Systems: The Case of Spike Protein of SARS-CoV-2 Virus.* . Computational and Structural Biotechnology Journal 2021. **19**: p. 1288-1301, DOI: 10.1016/j.csbj.2021.02.004.
4. Adhikari, P. and W.-Y. Ching, *Amino acid interacting network in the receptor-binding domain of SARS-CoV-2 spike protein.* RSC Advances 2020. **10**: p. 39831-39841, DOI: 10.1039/d0ra08222h.
5. Adhikari, P., N. Li, M. Shin, N.F. Steinmetz, R. Twarock, R. Podgornik, and W.-Y. Ching, *Intra- and intermolecular atomic-scale interactions in the receptor binding domain of SARS-CoV-2 spike protein: implication for ACE2 receptor binding.* Physical Chemistry Chemical Physics, 2020. **22**(33): p. 18272-18283, DOI: 10.1039/D0CP03145C.
6. Poudel, L., N.F. Steinmetz, R.H. French, V.A. Parsegian, R. Podgornik, and W.-Y. Ching, *Implication of the solvent effect, metal ions and topology in the electronic structure and hydrogen bonding of human telomeric G-quadruplex DNA.* Physical Chemistry Chemical Physics, 2016. **18**(31): p. 21573-21585, DOI: 10.1039/c6cp04357g.
7. Poudel, L., R. Twarock, N.F. Steinmetz, R. Podgornik, and W.-Y. Ching, *Impact of hydrogen bonding in the binding site between capsid protein and MS2 bacteriophage ssRNA.* The Journal of Physical Chemistry B, 2017. **121**(26): p. 6321-6330, DOI: 10.1021/acs.jpcb.7b02569.
8. Eifler, J., R. Podgornik, N.F. Steinmetz, R.H. French, V.A. Parsegian, and W.Y. Ching, *Charge distribution and hydrogen bonding of a collagen α2-chain in vacuum, hydrated, neutral, and charged structural models.* International Journal of Quantum Chemistry, 2016. **116**(9): p. 681-691, DOI: 10.1002/qua.25089.
9. Poudel, L., A.M. Wen, R.H. French, V.A. Parsegian, R. Podgornik, N.F. Steinmetz, and W.Y. Ching, *Electronic structure and partial charge distribution of doxorubicin in different molecular environments.* ChemPhysChem, 2015. **16**(7): p. 1451-1460, DOI: 10.1002/cphc.201402893.
10. Poudel, L., P. Rulis, L. Liang, and W.-Y. Ching, *Electronic structure, stacking energy, partial charge, and hydrogen bonding in four periodic B-DNA models.* Physical Review E, 2014. **90**(2): p. 022705, DOI: 10.1103/PhysRevE.90.022705.
11. Adhikari, P., M. Xiong, N. Li, X. Zhao, P. Rulis, and W.-Y. Ching, *Structure and electronic properties of a continuous random network model of an amorphous zeolitic imidazolate framework (a-ZIF).* The Journal of Physical Chemistry C, 2016. **120**(28): p. 15362-15368, DOI: 10.1021/acs.jpcc.6b06337.
12. Ching, W.Y., M. Yoshiya, P. Adhikari, P. Rulis, Y. Ikuhara, and I. Tanaka, *First-principles study in an inter-granular glassy film model of silicon nitride.* Journal of the American Ceramic Society, 2018. **101**(7): p. 2673-2688, DOI: 10.1111/jace.15538.

13. Ching, W.-Y., S. San, J. Brechtl, R. Sakidja, M. Zhang, and P.K. Liaw, *Fundamental electronic structure and multiatomic bonding in 13 biocompatible high-entropy alloys.* npj Computational Materials, 2020. **6**(1): p. 1-10, DOI: 10.1038/s41524-020-0321-x.
14. Jawad, B., L. Poudel, R. Podgornik, and W.-Y. Ching, *Thermodynamic Dissection of the Intercalation Binding Process of Doxorubicin to dsDNA with Implications of Ionic and Solvent Effects.* The Journal of Physical Chemistry B, 2020. **124**(36): p. 7803-7818, DOI: 10.1021/acs.jpcb.0c05840.
15. Baral, K., A. Li, and W.-Y. Ching, *Ab Initio Study of Hydrolysis Effects in Single and Ion-Exchanged Alkali Aluminosilicate Glasses.* The Journal of Physical Chemistry B, 2020. **124**(38): p. 8418-8433, DOI: 10.1021/acs.jpcb.0c05875.
16. Mulliken, R.S., *Electronic population analysis on LCAO–MO molecular wave functions. I.* The Journal of Chemical Physics, 1955. **23**(10): p. 1833-1840, DOI: 10.1063/1.1740588.
17. Mulliken, R., *Electronic population analysis on LCAO–MO molecular wave functions. II. Overlap populations, bond orders, and covalent bond energies.* The Journal of Chemical Physics, 1955. **23**(10): p. 1841-1846, DOI: 10.1063/1.1740589.
